# Knowledge, Attitudes, and Behaviors Toward Salt Consumption and Its Association With 24-Hour Urinary Sodium and Potassium Excretion in Adults Living in Mexico City: Cross-Sectional Study

**DOI:** 10.2196/57265

**Published:** 2024-11-18

**Authors:** Gabriela Gutiérrez-Salmeán, Paola Vanessa Miranda-Alatriste, Patricio Benítez-Alday, Luis Enrique Orozco-Rivera, Nurit Islas-Vargas, Ángeles Espinosa-Cuevas, Ricardo Correa-Rotter, Eloisa Colin-Ramirez

**Affiliations:** 1 Centro de Investigación en Ciencias de la Salud Facultad de Ciencias de la Salud Universidad Anáhuac México Huixquilucan, Estado de México Mexico; 2 Servicio de Nutrición Centro de Especialidades del Riñón Ciudad de México Mexico; 3 Departamento de Nefrología y Metabolismo Mineral Instituto Nacional de Ciencias Médicas y Nutrición Salvador Zubirán Ciudad de México Mexico; 4 Dirección de Nutrición Instituto Nacional de Ciencias Médicas y Nutrición Salvador Zubirán Ciudad de México Mexico

**Keywords:** beliefs, attitudes, hypertension, knowledge, salt consumption, sodium intake, potassium intake, Mexico

## Abstract

**Background:**

The World Health Organization recommends a daily sodium intake of less than 2000 mg for adults; however, the Mexican population, like many others globally, consumes more sodium than this recommended amount. Excessive sodium intake is often accompanied by inadequate potassium intake. The association between knowledge, attitudes, and behaviors (KAB) and actual sodium intake has yielded mixed results across various populations. In Mexico, however, salt/sodium-related KAB and its relationship with sodium and potassium intake have not been evaluated.

**Objective:**

This study primarily aims to describe salt/sodium-related KAB in a Mexican population and, secondarily, to explore the association between KAB and 24-hour urinary sodium and potassium excretion.

**Methods:**

We conducted a cross-sectional study in an adult population from Mexico City and the surrounding metropolitan area. Self-reported KAB related to salt/sodium intake was assessed using a survey developed by the Pan American Health Organization. Anthropometric measurements were taken, and 24-hour urinary sodium and potassium excretion levels were determined. Descriptive statistics were stratified by sex and presented as means (SD) or median (25th-75th percentiles) for continuous variables, and as absolute and relative frequencies for categorical variables. The associations between KAB and sodium and potassium excretion were assessed using analysis of covariance, adjusting for age, sex, BMI, and daily energy intake as covariates, with the Šidák correction applied for multiple comparisons.

**Results:**

Overall, 232 participants were recruited (women, n=184, 79.3%). The mean urinary sodium and potassium excretion were estimated to be 2582.5 and 1493.5 mg/day, respectively. A higher proportion of men did not know the amount of sodium they consumed compared with women (12/48, 25%, vs 15/184, 8.2%, P=.01). More women reported knowing that there is a recommended amount for daily sodium intake than men (46/184, 25%, vs 10/48, 20.8%, P=.02). Additionally, more than half of men (30/48, 62.5%) reported never or rarely reading food labels, compared with women (96/184, 52.1%, P=.04). Better salt/sodium-related KAB was associated with higher adjusted mean sodium and potassium excretion. For example, mean sodium excretion was 3011.5 (95% CI 2640.1-3382.9) mg/day among participants who reported knowing the difference between salt and sodium, compared with 2592.8 (95% CI 2417.2-2768.3) mg/day in those who reported not knowing this difference (P=.049). Similarly, potassium excretion was 1864.9 (95% CI 1669.6-2060.3) mg/day for those who knew the difference, compared with 1512.5 (95% CI 1420.1-1604.8) mg/day for those who did not (P=.002). Additionally, higher urinary sodium excretion was observed among participants who reported consuming too much sodium (3216.0 mg/day, 95% CI 2867.1-3565.0 mg/day) compared with those who claimed to eat just the right amount (2584.3 mg/day, 95% CI 2384.9-2783.7 mg/day, P=.01).

**Conclusions:**

Salt/sodium-related KAB was poor in this study sample. Moreover, KAB had a greater impact on potassium excretion than on sodium excretion, highlighting the need for more strategies to improve KAB related to salt/sodium intake. Additionally, it is important to consider other strategies aimed at modifying the sodium content of foods.

## Introduction

Cardiovascular diseases (CVDs) are the leading cause of death worldwide, with hypertension being one of the primary contributors, affecting over 20% of the Mexican adult population [1]. Excessive sodium intake is recognized as a major diet-related risk factor for hypertension; thus, reducing dietary sodium has been identified as a cornerstone in medical nutritional therapy for managing hypertension and other CVDs [2]. The World Health Organization (WHO) recommends a daily sodium intake of up to 2000 mg [3]. However, the adult Mexican population has been reported to consume 1.5 times this amount, averaging between 3100 and 3500 mg per day [1,2,4]. Notably, high sodium intake is often accompanied by low potassium intake [1,2,4], which is considered an indicator of poor diet quality [5]. Additionally, the combination of high sodium and low potassium intake is linked to an increased cardiovascular risk in a dose-response relationship [6].

Several policies and campaigns have been implemented to support sodium reduction at the population level, including labeling products high in sodium for easier identification and removing table salt from restaurant tables. Preventable risk models indicate that reducing salt/sodium consumption could prevent a significant number of deaths from CVDs [7].

By contrast, patients’ beliefs and perceived knowledge are strong predictors of their attitudes (ie, actions) and may, therefore, directly impact adherence to medical nutrition therapy [8,9]. Knowledge is defined as the understanding of a subject or topic, including the cognitive ability to retain such information [10]. Attitudes refer to the emotional, motivational, perceptual, and cognitive beliefs that positively or negatively influence a person’s behavior. They affect future behavior independently of an individual’s knowledge, helping to explain, at least in part, why a person adopts one behavior over others [10]. Finally, behaviors are defined as a set of responses of an individual or organism to external stimuli or internal motivation [11].

Although people may be aware of the health implications of high salt or sodium intake, their knowledge, attitudes, and behaviors (KAB) on this issue can still be lacking, as shown in a systematic review of 24 studies across 12 high-income countries [12]. Moreover, studies on the association between KAB and actual sodium intake have yielded mixed results in diverse populations; some studies report no association between salt- or sodium-related KAB and sodium excretion, while others have found certain associations [13-20]. Additionally, the relationship between KAB and potassium intake has received less attention [15]. In Mexico, salt- and sodium-related KAB and its association with sodium and potassium intake have not yet been studied.

Given this context, we aimed to describe self-reported KAB related to salt and sodium consumption in a sample of volunteers from Mexico City and the metropolitan area. Additionally, we explored the association between salt- and sodium-related KAB and 24-hour urinary sodium and potassium excretion as a surrogate for actual intake.

## Methods

### Study Design

This is a secondary analysis of data from a cross-sectional study designed to test the criterion validity of an online tool [21] for estimating sodium intake in the adult population. The validation results are currently under review for publication elsewhere.

### Study Population and Setting

The original study included men and women over 18 years of age who were apparently healthy. Exclusion criteria were individuals over 65 years; those with a previous diagnosis of CVD, kidney disease, or liver disease; and those who were menstruating, pregnant, or had urinary tract infections at the time of 24-hour urine collection. A convenience sample of participants meeting the eligibility criteria was recruited from various workplaces in Mexico City and the metropolitan area from June 2022 to January 2023. Participants were invited to join through printed advertisements on bulletin boards and a word-of-mouth strategy. Once eligibility was confirmed, they received both verbal and written instructions to collect a 24-hour urine sample and maintain a 3-day food record, which they were to bring on the day of their study visit. All assessments took place at designated areas within each work center.

For this secondary analysis, participants who provided a complete 24-hour urine sample and completed the salt/sodium-related KAB survey were included.

### Ethical Considerations

This study posed no risk to participants, and their data were handled with privacy and confidentiality. Participants’ data were deidentified for analysis, with only the principal investigator having access to the identified data. The study was approved by the Research and Ethics Board of the Instituto Nacional de Ciencias Médicas y Nutrición, under registration number 3314. All participants were fully informed about the study, and written informed consent was obtained. No compensation was offered to participants for their involvement in this study, other than the provision of the results from the body composition assessment conducted as part of the original study, as well as the results of the urinary analysis.

### Assessments

#### Anthropometrics

Weight and height were measured with the participant fasting, without shoes, and wearing a hospital gown, following the guidelines established by the International Society for the Advancement of Kinanthropometry (ISAK) [22]. Weight was measured using a Seca 769 mechanical column scale with a capacity of 200 kg and a precision of 0.05 kg. Height was measured with a Seca 220 stadiometer with a precision of 1 mm. BMI was calculated and reported as kg/m^2^.

#### 24-Hour Urinary Sodium and Potassium

Participants were given both oral and written instructions to collect a 24-hour urine sample the day before the study visit. They were instructed to discard the first-morning void and collect all urine over the following 24 hours, including the first void of the next morning, using a preservative-free container. Participants were also instructed to store the collected urine in a cool place during the collection period. Twenty-four-hour urinary excretion of sodium, potassium, and creatinine was determined.

Urinary samples were considered complete if they met the creatinine excretion rate criteria: 15-25 mg/kg/24 hours for men and 10-20 mg/kg/24 hours for women [23]. Additionally, urine samples with a volume of less than 500 ml per 2 hours were considered incomplete.

#### Energy Intake

Participants were instructed to complete a 3-day food record, including 1 weekend day. On the day of the study visit, a member of the research team verified the completeness and clarity of the food record with the participant. Dietary information was collected by trained personnel using a nutrient analysis software program (ESHA Food Processor SQL version 10.11; ESHA Research). The mean energy intake from the 3 days was used for analysis.

#### Knowledge, Attitudes, and Behaviors

A self-administered questionnaire developed by a subgroup of the Pan American Health Organization (PAHO) expert group, tasked with examining excessive dietary salt as a health risk in the Americas, was used. This tool was designed to assess salt-related knowledge (eg, the difference between sodium and salt), attitudes (eg, concern about reducing salt consumption), and behavior (eg, frequency of adding salt to food during cooking and at the table), self-reported presence of chronic diseases, and labeling preferences. The questionnaire was field-tested in Latin America and Canada. The questions included in this tool were developed based on the experience and expertise of the expert group members, as well as questions used in other surveys [24].

Participants received guidance from research team members to clarify any unclear questions as needed to complete the questionnaire.

Questions regarding labeling preferences (eg, whether participants would like food labeling indicating high/medium/low levels of salt or sodium) were not included in the analysis, as they were beyond the scope of this study.

#### Other Data

Information on age, sex, and highest level of education was also collected.

### Statistical Analysis

Descriptive statistics were stratified by sex and reported as mean (SD) or median (25th-75th percentiles) for continuous variables, based on their distribution as determined by the Kolmogorov-Smirnov test. Categorical variables were presented as absolute frequencies and proportions. Comparisons of KAB related to health and salt/sodium consumption categories between sex groups were made using the chi-square test. In addition, one-way fixed-effects analyses of covariance were used to compare the adjusted means of 24-hour urinary sodium and potassium excretion across the KAB related to salt/sodium consumption categories (groups) for each KAB item. Separate models were constructed for 24-hour urinary sodium and potassium excretion. In these models, urinary sodium and potassium excretion were treated as the dependent variables, with each relevant item of salt/sodium-related KAB tested separately as the independent variable (factor) with multiple categories (groups). Factors known to be associated with sodium and potassium intake (age, sex, BMI, and daily energy intake [DEI]) [2,4] were included as covariates in all models. The general linear model used to compare 24-hour urinary sodium and potassium excretion among salt/sodium-related KAB groups, adjusting for the effect of covariates, can be expressed as follows [25,26]:

24-hour UNa*_ij_*=μ + α*_i_* + β_1_age*_ij_* + β_2_sex*_ij_* + β_3_BMI*_ij_* + β_4_DEI*_ij_* + *e_ij_*

24-hour UK*_ij_*=μ + α*_i_* + β_1_age*_ij_* + β_2_sex*_ij_* + β_3_BMI*_ij_* + β_4_DEI*_ij_* + *e_ij_*

where 24-h UNa*_ij_* is the 24-hour urinary sodium excretion of the *j*th participant in the *i*th salt/sodium-related KAB group, 24-h UK*_ij_* is the 24-hour urinary potassium excretion of the *j*th participant in the *i*th salt/sodium-related KAB group, μ is a grand mean, a*_i_* is the *i*th group effect, β_1_ is the slope coefficient of age, β_2_ is the slope coefficient of sex, β_3_ is the slope coefficient of BMI, β_4_ is the slope coefficient of DEI, and *e_ij_* is the residual.

95% CIs were constructed for the adjusted means, and the Šidák correction was applied for multiple comparisons. All statistical analyses were performed using SPSS version 20 for Windows (IBM Corp.). A *P* value of <.05 was considered statistically significant.

## Results

### Study Sample Descriptive Characteristics

Overall, 365 participants were recruited. Of these, 232 completed the salt/sodium-related KAB questionnaire and provided a valid 24-hour urinary sodium sample; these participants were included in this subanalysis. Study sample characteristics by sex are shown in Table 1. The majority of the study sample were women (184/232, 79.3%), with a median age of 39.0 (25th-75th percentiles 27.3-49.0) years and a BMI of 25.8 (25th-75th percentiles 22.9-29.2) kg/m^2^ for the overall population. In terms of education, most participants had completed an education level higher than secondary (101/232, 43.5%). The median energy intake was estimated to be 2704.7 (25th-75th percentiles 2128.0-3271.1) kcal/day. Estimates of urinary sodium, potassium, and creatinine are also shown in Table 1.

**Table 1:** Study sample characteristics by sex^a^.

Characteristics		Overall (n=232)	Women (n=184)	Men (n=48)
Age (years)		39.0 (27.3-49.0)	41 (29-50)	36.9 (12.5)
Weight (kg)		63.7 (55.5-71.8)	62.7 (12.3)	70.8 (62.4-80.4)
Height (cm)		156.0 (150-162.8)	154.0 (6.8)	167.1 (8.2)
BMI (kg/m^2^)		25.8 (22.9-29.2)	26.4 (5.0)	25.6 (23.6-27.4)
**Education level**				
	None	6 (2.6)	6 (3.3)	0 (0)
	Primary	29 (12.5)	25 (13.6)	4 (8.3)
	Secondary	93 (40.1)	72 (39.1)	21 (43.8)
	Higher	101 (43.5)	79 (42.9)	22 (45.8)
	No answer	3 (1.3)	2 (1.1)	1 (2.1)
Energy intake (kcal/day)		2704.7 (2128.0-3271.1)	2726.6 (2141.5-3254.4)	2778.2 (981.8)
Diuresis (ml/24 hours)		1225.0 (900.0-1700.0)	1200 (900-1587.5)	1400.0 (1000.0-2000.0)
Sodium (mg/24 hours)		2582.5 (1701.6-3347.2)	2570.4 (1222.3)	2845.1 (2231.4-3631.1)
Potassium (mg/24 hours)		1493.5 (1096.3-1934.7)	1431.9 (1071.0-1894.5)	1873.2 (736.4)
Sodium-to-potassium ratio (mg/mg/24 hours)		1.7 (1.3-2.3)	1.7 (1.3-2.3)	1.6 (1.3-2.1)
Creatinine (mg/24 hours)		87.1 (61.4-119.7)	81.0 (56.6-111.11)	108.8 (86.2-163.2)

^a^Numerical data are presented as mean (SD) or median (25th-75th percentiles), according to their distribution. Categorical data are presented as n (%).

Regarding the self-reported presence of chronic diseases included in the survey, 43 of the 232 (18.5%) participants reported having high blood pressure. Other pathologies associated with excessive salt consumption were also surveyed and are reported, stratified by sex, in Table 2.

**Table 2:** Self-reported history of chronic diseases by sex.

Disease	Overall (n=232)	Women (n=184)	Men (n=48)
High blood pressure, n (%)	43 (18.5)	40 (21.7)	3 (6.3)
Heart attack, n (%)	7 (3.0)	6 (3.3)	1 (2.1)
Stroke, n (%)	1 (0.4)	1 (0.5)	0 (0)
Kidney stones, n (%)	14 (6.0)	10 (5.4)	4 (8.3)
Asthma, n (%)	10 (4.3)	10 (5.4)	0 (0)
Osteoporosis, n (%)	2 (0.9)	2 (1.1)	0 (0)
Stomach cancer, n (%)	1 (0.4)	1 (0.5)	0 (0)

### Attitudes, Knowledge, and Behaviors

As shown in Table 3, more than half of the participants reported trying to eat a healthy diet and consistently feeling pressure to do so. Nearly the entire sample (229/232, 98.7%) acknowledged that excessive salt is associated with health problems, although only two-thirds believed they were capable of distinguishing between products low or high in salt. Furthermore, less than half of the sample indicated that there was sufficient information on salt content in food packages. Notably, only 93 of the 232 (40.1%) participants reported overall good health, with a trend toward a lower proportion of women compared with men (71/184, 38.6%, vs 22/48, 45.8%, *P*=.07). The number of participants who reported trying to minimize the amount of salt they consumed was 174 (75%) in the overall population (N=232), and this tendency was higher among women than men (143/184, 77.7%, vs 31/48, 64.6%, *P*=.08). However, no significant differences were found between men and women in any of the questions (*P*≥.05; Table 3).

**Table 3:** The proportion of participants who agreed with the statement on self-reported attitudes, knowledge, and behavior related to health, diet, and salt intake in the study sample.

Statement	Overall (n=232)	Women (n=184)	Men (n=48)	*P* value^a^
I try to eat a healthy diet, n (%)	153 (65.9)	120 (65.2)	33 (68.8)	.71
A diet high in salt can cause serious health problems, n (%)	229 (98.7)	182 (98.9)	47 (97.9)	.52
I try to minimize the amount of fat I consume, n (%)	187 (80.6)	149 (81.0)	38 (79.2)	.68
My health is generally good, n (%)	93 (40.1)	71 (38.6)	22 (45.8)	.07
There is too much pressure to eat healthy these days, n (%)	134 (57.8)	109 (59.2)	25 (52.1)	.76
I try to minimize the amount of salt I consume, n (%)	174 (75.0)	143 (77.7)	31 (64.6)	.08
I generally know if foods contain a lot or little salt, n (%)	148 (63.8)	122 (66.3)	26 (54.2)	.12
There is sufficient nutritional information on food packaging, n (%)	106 (45.7)	84 (45.7)	22 (45.8)	.34

^a^For differences between sex groups by chi-square test.

Additionally, 150 of the 232 (64.7%) participants reported always adding salt when preparing food at home, while more than 90% considered limiting the amount of salt or sodium in their diet to be very or somewhat important (210/232, 90.5%). Around 60% believed they consumed the right amount of salt (139/232, 59.9%), yet less than a quarter knew there was a recommended daily amount of sodium consumption; 186 of 232 (80.2%) participants did not know the difference between salt and sodium, and over half reported rarely or never reading nutrition labels on food packages. Comparison between sex groups revealed a higher proportion of men who did not know the amount of sodium they consumed compared with women (12/48, 25%, vs 15/184, 8.2%, *P*=.01), while more women reported knowing that there was a recommended daily sodium intake than men (46/184, 25%, vs 10/48, 20.8%, *P*=.02). Notably, there was a difference in the frequency of reading nutrition labels on food packages between sex groups, with more than half of men (30/48, 62.5%) reporting that they never or rarely read food labels, compared with women (96/184, 52.1%, *P*=.04; Table 4).

**Table 4:** Answers to questions about knowledge, attitudes, and behaviors regarding sodium intake.

Question		Overall (n=232)	Women (n=184)	Men (n=48)	*P* value^a^	
**How many times do you add salt to food at the table?, n (%)**					.12	
	Never	40 (17.2)	32 (17.4)	8 (16.7)		
	Rarely	102 (44.0)	85 (46.2)	17 (35.4)		
	Sometimes	58 (25.0)	45 (24.5)	13 (27.1)		
	Often	20 (8.6)	16 (8.7)	4 (8.3)		
	Always	12 (5.2)	6 (3.3)	6 (12.5)		
**Do you add salt when preparing food at home?, n (%)**					.12	
	Never	7 (3.0)	3 (1.6)	4 (8.3)		
	Rarely	12 (5.2)	10 (5.4)	2 (4.2)		
	Sometimes	24 (10.3)	17 (9.2)	7 (14.6)		
	Often	39 (16.8)	32 (17.4)	7 (14.6)		
	Always	150 (64.7)	122 (66.3)	28 (58.3)		
**Is limiting the amount of salt or sodium in your diet important to you?, n (%)**					.23	
	Very	95 (40.9)	77 (41.8)	18 (37.5)		
	Somewhat	115 (49.6)	93 (50.5)	22 (45.8)		
	No	14 (6.0)	8 (4.3)	6 (12.5)		
	Do not know	6 (2.6)	4 (2.2)	2 (4.2)		
	No answer	2 (0.9)	2 (1.1)	0 (0)		
**How much salt do you think you consume?, n (%)**					.01	
	Not enough	18 (7.8)	16 (8.7)	2 (4.2)		
	Right amount	139 (59.9)	111 (60.3)	28 (58.3)		
	Too much	46 (19.8)	40 (21.7)	6 (12.5)		
	Do not know	27 (11.6)	15 (8.2)	12 (25.0)		
	No answer	2 (0.9)	2 (1.1)	0 (0)		
**Do you know if there is a recommended amount of salt/sodium consumption per person per day?, n (%)**					.02	
	Yes	56 (24.1)	46 (25.0)	10 (20.8)		
	No	174 (75.0)	138 (75.0)	36 (75.0)		
	No answer	2 (0.9)	0 (0)	2 (4.2)		
**Do you know the difference between salt and sodium?, n (%)**					.42	
	Yes	44 (19.0)	33 (17.9)	11 (22.9)		
	No	186 (80.2)	150 (81.5)	36 (75.0)		
	No answer	2 (0.9)	1 (0.5)	1 (2.1)		
**Do you pay attention to the text on the packaging such as no added salt, low salt, light, trans-fat free?, n (%)**					.23	
	Always	12 (5.2)	9 (4.9)	3 (6.3)		
	Often	35 (15.1)	31 (16.8)	4 (8.3)		
	Sometimes	63 (27.2)	53 (28.8)	10 (20.8)		
	Rarely	67 (28.9)	53 (28.8)	16 (33.3)		
	Never	49 (21.1)	37 (20.1)	12 (25.0)		
	Do not know	6 (2.6)	3 (1.6)	3 (6.3)		
**How often do you read nutrition labels on food packages?, n (%)**					.04	
	Always	12 (5.2)	8 (4.3)	4 (8.3)		
	Often	26 (11.2)	20 (10.9)	6 (12.5)		
	Sometimes	67 (28.9)	60 (32.6)	7 (14.6)		
	Rarely	91 (39.2)	72 (39.1)	19 (39.6)		
	Never	35 (15.1)	24 (13.0)	11 (22.9)		
	No answer	1 (0.4)	0 (0)	1 (2.1)		

^a^For differences between sex groups by chi-square test.

### Salt/Sodium-Related Attitudes, Knowledge, and Behaviors and 24-Hour Urinary Sodium and Potassium Excretion

A comparison of the adjusted means (95% CI) for 24-hour urinary sodium and potassium excretion across categories of salt/sodium-related KAB is shown in Table 5. A higher mean 24-hour urinary sodium excretion was observed among participants who reported consuming too much sodium, compared with those who believed they consumed the right amount. It was also higher among participants who knew that there was a recommended daily sodium intake and who understood the difference between salt and sodium, compared with those who did not. Additionally, participants who frequently paid attention to the text on food packaging had a higher mean urinary sodium excretion than those who rarely did so. Although the overall comparison for the frequency of reading nutrition labels showed a significant difference among frequency categories (*P*=.02), pairwise comparisons did not reveal any significant differences (*P*≥.05 for all pairwise comparisons).

Regarding urinary potassium excretion, participants who tried to eat a healthy diet had a higher mean potassium excretion compared with those who were unsure if they were trying to eat a healthy diet. Additionally, although a significant difference in potassium excretion was observed in the overall comparison among categories of the amount of salt participants thought they consumed (*P*=.02), pairwise comparisons did not reveal any significant differences (*P*≥.05 for all pairwise comparisons). Higher potassium excretion was also observed among participants who knew there was a recommended daily sodium intake and those who claimed to know the difference between salt and sodium, compared with those who did not. In terms of reading food labels, participants who reported often paying attention to the text on food packaging had greater potassium excretion compared with those who rarely did so. Similarly, individuals who always read nutrition food labels had higher potassium excretion compared with those who sometimes, rarely, or never read food labels (Table 5).

**Table 5:** Comparison of urinary sodium and potassium excretion adjusted means (95% CI) among groups within the items of knowledge, attitudes, and behaviors regarding sodium intake.

Question		24-h urinary sodium excretion (mg/day), adjusted means (95% CI)	24-h urinary potassium excretion (mg/day), adjusted means (95% CI)
**I try to eat a healthy diet**			
	Agree (n=153)	2802.9 (2606.3-2999.6)	1666.5 (1561.1-1771.9)^a^
	Disagree (n=31)	2396.6 (1956.7-2836.4)	1557.2 (1321.4-1793.0)
	Do not know (n=35)	2576.2 (2164.1-2988.3)	1331.5 (1110.5-1552.4)^a^
	*P* value	.21	.03
**A diet high in salt can cause serious health problems**			
	Agree (n=229)	2696.7 (2538.1-2855.4)	1594.6 (1508.6-1680.5)
	Do not know (n=2)	1900.0 (195.2-3604.7)	1530.6 (607.2-2454.0)
	*P* value	.36	.89
**I try to minimize the amount of salt I consume**			
	Yes (n=174)	2732.0 (2549.9-2914.2)	1603.7 (1504.3-1703.2)
	No (n=40)	2416.5 (2035.4-2797.6)	1528.7 (1320.6-1736.8)
	Do not know (n=13)	2598.1 (1927.7-3268.4)	1631.8 (1265.7-1997.8)
	*P* value	.34	.80
**I generally know if foods contain a lot or little salt**			
	Yes (n=148)	2744.3 (2545.4-2943.2)	1636.3 (1528.7-1743.8)
	No (n=80)	2608.4 (2336.8-2880.1)	1517.4 (1370.5-1664.3)
	*P* value	.43	.20
**How many times do you add salt to food at the table?**			
	Never (n=40)	2766.4 (2384.4-3148.5)	1624.1 (1416.5-1831.7)
	Rarely (n=102)	2686.9 (2446.9-2926.9)	1572.0 (1441.5-1702.4)
	Sometimes (n=58)	2638.2 (2320.4-2956.0)	1570.5 (1397.9-1743.2)
	Often (n=20)	2897.6 (2354.7-3440.6)	1630.4 (1335.4-1925.5)
	Always (n=12)	2221.2 (1514.6-2927.8)	1663.1 (1279.2-2047.1)
	*P* value	.64	.98
**Do you add salt when preparing food at home?**			
	Never (n=7)	2561.7 (1637.7-3485.6)	1943.3 (1437.9-2448.7)
	Rarely (n=12)	3656.2 (2966.3-4346.0)	1748.9 (1371.6-2126.3)
	Sometimes (n=24)	2694.3 (2206.9-3181.7)	1478.5 (1211.9-1745.1)
	Often (n=39)	2709.1 (2321.9-3096.3)	1586.4 (1374.6-1798.2)
	Always (n=150)	2601.5 (2405.8-2797.1)	1580.1 (1473.1-1687.1)
	*P* value	.08	.51
**How much salt do you think you consume?**			
	Not enough (n=18)	2346.7 (1788.3-2905.0)	1396.8 (1090.9-1702.8)
	Right amount (n=139)	2584.3 (2384.9-2783.7)^b^	1550.0 (1440.7-1659.2)
	Too much (n=46)	3216.0 (2867.1-3565.0)^b^	1845.3 (1654.1-2036.5)
	Do not know (n=27)	2471.7 (2000.1-2943.2)	1484.5 (1226.2-1742.9)
	*P* value	.008	.02
**Is limiting the amount of salt or sodium in your diet important to you?**			
	Very (n=95)	2800.6 (255.8-3050.3)	1558.6 (1423.7-1693.4)
	Somewhat (n=115)	2630.5 (2405.1-2855.9)	1632.0 (1510.3-1753.7)
	No (n=14)	2373.4 (1712.3-3034.5)	1525.4 (1168.5-1882.3)
	Do not know (n=6)	3005.0 (2017-3993.0)	1756.7 (1223.4-2290.1)
	*P* value	.52	.77
**Do you know if there is a recommended amount of salt/sodium consumption per person per day?**			
	Yes (n=56)	3056.7 (2733.1-3380.2)	1882.5 (1711.4-2053.6)
	No (n=174)	2562.9 (2381.5-2744.2)	1488.3 (1392.3-1584.2)
	*P* value	.01	<.001
**Do you know the difference between salt and sodium?**			
	Yes (n=44)	3011.5 (2640.1-3382.9)	1864.9 (1669.6-2060.3)
	No (n=186)	2592.8 (2417.2-2768.3)	1512.5 (1420.1-1604.8)
	*P* value	.049	.002
**Do you pay attention to the text on the packaging such as no added salt, low salt, light, trans-fat free?**			
	Always (n=12)	3034.4 (2348.8-3720.1)	1673.2 (1305.1-2041.4)
	Often (n=35)	3278.0 (2870.2-3685.8)^c^	1859.5 (1640.6-2078.5)^d^
	Sometimes (n=63)	2571.7 (2270.4-2873.0)	1692.0 (1530.2-1853.7)
	Rarely (n=67)	2501.3 (2210.5-2792.1)^c^	1390.7 (1234.5-1546.8)^d^
	Never (n=49)	2607.0 (2257.2-2956.7)	1559.3 (1371.5-1747.1)
	Do not know (n=6)	2308.9 (1332.1-3285.6)	1269.7 (745.3-1794.2)
	*P* value	.04	.01
**How often do you read nutrition labels on food packages?**			
	Always (n=12)	3456.4 (2764.0-4148.9)	2158.3 (1789.7-2526.9)^e,f,g^
	Often (n=26)	3214.7 (2739.9-3689.4)	1921.1 (1668.4-2173.8)^h^
	Sometimes (n=67)	2608.5 (2313.5-2903.6)	1560.7 (1403.6-1717.7)^e^
	Rarely (n=91)	2519.5 (2270.8-2768.1)	1473.4 (1341.0-1605.7)^f,h^
	Never (n=35)	2583.2 (2170.2-2996.3)	1509.9 (1290.0-1729.8)^g^
	*P* value	.02	.001

^a^*P*=.02; ^b^*P*=.01; ^c^*P*=.04; ^d^*P*=.01; ^e^*P*=.04; ^f^*P*=.007; ^g^*P*=.03; ^h^*P*=.02 for multiple comparisons by the Šidák test. Differences are between categories that have the same letter. All means were adjusted by sex, age, BMI, and daily energy intake by analysis of covariance.

## Discussion

### Principal Findings and Comparison With Prior Work

Based on the data collected and analyzed in this study, several important findings can be reported regarding consumers’ attitudes, knowledge, and behavior concerning salt intake, and their association with urinary sodium and potassium excretion.

According to the self-reported KAB related to health, diet, and salt consumption, among the total participants (N=232), only 56 (24.1%) knew there was a recommended daily intake for salt/sodium, 44 (19%) understood the difference between salt and sodium, and only 12 (5.2%) always paid attention to the text on food packaging or read nutrition labels. Importantly, a high proportion of participants reported trying to maintain a healthy diet (153/232, 65.9%) and reduce the amount of fats (187/232, 80.6%) or salt (174/232, 75%) they consumed. However, these responses did not align with their perception of health, as only 93 (40.1%) considered their health to be generally good. It is likely that participants were making these dietary changes in an effort to improve their health, which they perceived as poor.

In our study, we observed a trend where a smaller proportion of women (71/184, 38.6%) compared with men (22/48, 45.8%) reported having good health. Additionally, self-reported history of comorbidities related to excessive sodium intake revealed that 40 of 184 (21.7%) women and 3 of 48 (6.3%) men had high blood pressure. This lower perception of good health and higher frequency of self-reported high blood pressure among women is consistent with the observed trend of a higher proportion of women trying to minimize their salt consumption. Overall, studies that disaggregate KAB by sex have reported better KAB related to salt/sodium consumption in women than in men. In this study, we found that more women (46/184, 25.0%) reported knowing that there was a recommended daily sodium/salt intake compared with men (10/48, 20.8%). Additionally, more men (30/48, 62.5%) reported never or rarely reading food labels compared with women (96/184, 52.2%).

In the overall study sample, the median 24-hour urinary sodium excretion was 2582.5 mg/day, which exceeds the WHO daily intake recommendation of 2000 mg/day [3], but is lower than previous reports in the Mexican population, where it ranged from 3100 to 3500 mg/day [1,2,4]. This discrepancy may be attributed to the fact that nearly 80% (n=184) of the participants in this study were women, and sodium intake has been shown to be, on average, lower in women than in men [2,4], as also confirmed in this study; 32 out of 232 (13.8%) reported always or often adding salt to food at the table; however, a higher proportion (189/232, 81.5%) of participants reported always or often adding salt during food preparation. In Mexico, the consumption of processed and ultra-processed foods contributes to 39% of total sodium intake in adults and 50% in school-age children [27]. Nevertheless, salt added during cooking remains an important source of sodium in the diet [27]. In fact, it has been reported that street food (*antojitos* in Spanish) and homemade-style meals (*comida corrida* in Spanish), which are unpackaged and unlabeled prepared foods, contain high levels of sodium, with many providing more than 25% of the daily sodium intake recommended by the WHO [28]. Thus, the use of table salt in food preparation remains a significant challenge in Mexico, as it does in other countries such as Brazil, China, Costa Rica, Guatemala, India, Japan, Mozambique, and Romania, where a large proportion of overall daily sodium intake comes from the discretionary use of salt [29].

In terms of potassium intake, the median urinary excretion was 1493.5 mg/day in the overall study sample, which is considerably lower than the WHO’s recommended intake of 3510 mg/day for adults to prevent chronic disease and improve health [30]. Low urinary potassium excretion was also reported in 2 studies of the Mexican population, with values ranging from 1909 to 1981 mg/day [2,4], while another report indicated a higher mean potassium intake (3401 mg/day) in this population, as determined by a 24-hour food recall [27]. Importantly, potassium intake, whether determined by urinary excretion or dietary analysis, has been found to be an indicator of diet quality [5,31] and is inversely associated with BMI, diastolic blood pressure, and heart rate [31]. Additionally, the combination of higher sodium and lower potassium intakes, as observed in this and other studies [2,4,27], is associated with an increased cardiovascular risk [6]. Therefore, dietary strategies aimed at modifying both sodium and potassium intakes are necessary for the Mexican population.

Diverse studies have reported no association between salt/sodium-related KAB [13,17,18,20] and sodium excretion, while others have found some level of association [14,15,19]. To explore the relationship between salt/sodium-related KAB and sodium and potassium intake, we compared the adjusted means of 24-hour urinary sodium and potassium excretion across categories of salt/sodium-related KAB. Interestingly, the mean sodium excretion was similar among participants, regardless of whether they were trying to eat a healthy diet. However, potassium excretion was higher among participants who reported trying to eat a healthy diet compared with those who were unsure, after adjusting for sex, age, BMI, and calorie intake. Similarly, participants who often paid attention to the text on packaging had higher levels of both sodium and potassium excretion compared with those who rarely did so. Potassium excretion was also higher in participants who always read nutrition food labels compared with those who did so less frequently, although this behavior was not associated with sodium excretion in the multiple comparisons. These results suggest that attempts to follow a healthy diet may impact the quality of the diet by likely incorporating more food sources of potassium, such as fruits, vegetables, and legumes, but not necessarily modifying the selection of foods based on their sodium content. Another possibility is that to reduce sodium intake, people may increase the consumption of low-sodium processed foods that contain hidden potassium in the form of additives, such as sodium-reduced meat and poultry products and industrialized bakery items [32,33]. Further research is needed to clarify these findings.

Knowing that there is a recommended amount of daily salt/sodium consumption or understanding the differences between salt and sodium was not associated with lower sodium intake either; in fact, participants who reported knowing this exhibited higher sodium and potassium excretion than those who did not. These results are relevant as they support the findings of a study in a Chinese population, where self-reported salt/sodium-related KAB had a smaller effect on sodium but a greater effect on potassium excretion [15].

The results of this study provide evidence for the need to continue improving KAB related to salt/sodium consumption at the population level. The implementation of education and communication campaigns is one of the strategies endorsed by the WHO to raise awareness about the health risks and dietary sources of salt, aiming to induce behavioral changes [34]. In Mexico, the “Less Salt, More Health” campaign was implemented in 2013 by the Ministry of Health. This communication strategy aimed to increase knowledge and educate the population about the benefits of reducing salt consumption. However, the impact of its implementation was not assessed [1]. In fact, this is the first study to report KAB related to salt/sodium intake in a Mexican population.

Worldwide, other strategies beyond communication campaigns that have shown the greatest impact on dietary sodium reduction at the population level include mandatory food reformulation, food labeling, and taxes [35]. In 2020, Mexico implemented a mandatory front-of-pack warning labeling system [36]. The reformulation of processed and ultra-processed foods that contain large amounts of sodium should be one of the next steps in Mexico’s sodium reduction strategy. While some work has been done in this area, further progress is needed in line with the WHO regional sodium benchmarks [1].

Finally, even though better salt/sodium-related KAB was not associated with lower sodium excretion, participants had some awareness of their actual sodium intake level. Specifically, those who reported consuming the right amount of dietary sodium had lower sodium excretion compared with those who believed they consumed too much salt. Nonetheless, participants who reported an adequate intake (the right amount) still had sodium consumption levels above the recommended 2000 mg/day. This underscores the ongoing need to improve salt/sodium-related KAB and further reduce sodium intake, as previously discussed.

### Limitations

This study has several limitations. First, its cross-sectional design prevents the establishment of causal relationships between salt/sodium-related KAB and urinary sodium and potassium excretion. Second, the study was conducted with a sample from Mexico City and its surrounding area, limiting the generalizability of the results to the national population. Third, nearly 80% (n=184) of the study sample were women; however, data were stratified by sex, and the adjusted urinary sodium and potassium means accounted for sex as a covariate. Fourth, the available data do not distinguish between potassium naturally occurring in foods and that added as an additive in processed foods. Further studies are needed to explore the sources of dietary potassium in the Mexican population and its impact on health-related outcomes. Finally, no sample size calculation was conducted; instead, all available data from participants meeting the eligibility criteria for this secondary analysis were analyzed. Longitudinal and properly powered studies exploring this association are warranted. The main strength of this study lies in the use of an objective method to assess sodium and potassium intake, namely, the measurement of their excretion levels in 24-hour urine.

### Conclusions

Results of this study indicate that self-reported KAB related to salt/sodium intake is poor in this study sample. Therefore, new initiatives are needed to improve KAB in order to promote better food choices, which, in turn, could help reduce sodium intake. In this context, educating individuals about the sodium content of unpackaged prepared foods is particularly relevant. In addition, salt/sodium-related KAB had a greater impact on potassium excretion than on sodium excretion, highlighting the need for more strategies to improve KAB related to salt/sodium intake. It also emphasizes the importance of considering other approaches to modify the sodium content of foods, such as the reformulation of processed and ultra-processed foods. Finally, monitoring KAB related to salt/sodium consumption, along with actual sodium intake, in larger populations is essential, especially when new strategies to reduce sodium intake are implemented.

## Abbreviations

CVD: cardiovascular disease
DEI: daily energy intake
ISAK: International Society for the Advancement of Kinanthropometry
KAB: knowledge, attitudes, and behaviors
PAHO: Pan American Health Organization
WHO: World Health Organization

## Data Availability

The data sets generated and analyzed during this study are available from the corresponding author upon reasonable request.
